# E3-Specific Degrader Discovery by Dynamic Tracing of Substrate Receptor Abundance

**DOI:** 10.1021/jacs.2c10784

**Published:** 2023-01-05

**Authors:** Alexander Hanzl, Eleonora Barone, Sophie Bauer, Hong Yue, Radosław P. Nowak, Elisa Hahn, Eugenia V. Pankevich, Anna Koren, Stefan Kubicek, Eric S. Fischer, Georg E. Winter

**Affiliations:** † CeMM Research Center for Molecular Medicine of the Austrian Academy of Sciences, 1090 Vienna, Austria; ‡ Department of Cancer Biology, Dana-Farber Cancer Institute, Boston, Massachusetts 02215, United States; § Department of Biological Chemistry and Molecular Pharmacology, Harvard Medical School, Boston, Massachusetts 02115, United States

## Abstract

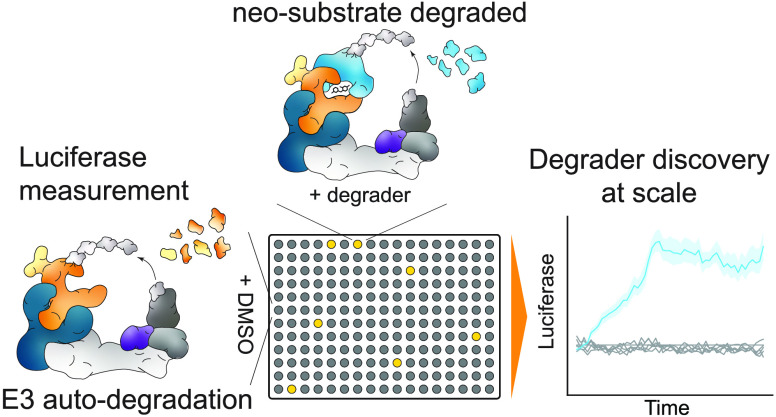

Targeted protein degradation (TPD) is a new pharmacology based on small-molecule degraders that induce proximity between a protein of interest (POI) and an E3 ubiquitin ligase. Of the approximately 600 E3s encoded in the human genome, only around 2% can be co-opted with degraders. This underrepresentation is caused by a paucity of discovery approaches to identify degraders for defined E3s. This hampers a rational expansion of the druggable proteome and stymies critical advancements in the field, such as tissue- and cell-specific degradation. Here, we focus on dynamic NEDD8 conjugation, a post-translational, regulatory circuit that controls the activity of 250 cullin RING E3 ligases (CRLs). Leveraging this regulatory layer enabled us to develop a scalable assay to identify compounds that alter the interactome of an E3 of interest by tracing their abundance after pharmacologically induced auto-degradation. Initial validation studies are performed for CRBN and VHL, but proteomics studies indicate broad applicability for many CRLs. Among amenable ligases, we select CRL^DCAF15^ for a proof-of-concept screen, leading to the identification of a novel DCAF15-dependent molecular glue degrader inducing the degradation of RBM23 and RBM39. Together, this strategy empowers the scalable identification of degraders specific to a ligase of interest.

## Introduction

Modulation of proximity between macromolecules is a central regulatory layer in most cellular processes. Chemically inducing proximity between two target proteins is an established mechanism of action of natural products as well as synthetic small molecules, and has expanded to clinical applications.^1−3^ In some instances, drug-induced proximity results in a functional inhibition of one participating protein via steric confinements imposed by the other effector. In other instances, the result of induced proximity can be an emerging effect where one protein partner is endowed with a novel, *neomorphic* function.^4^ This scenario is best illustrated by small-molecule degraders that recruit proteins of interest (POIs) to an E3 ligase. The change in E3 ligase interactome consequently leads to ubiquitination and degradation of the POI, which it would not recognize in the absence of the small molecule.^5^ This concept of targeted protein degradation (TPD) comes with several advantages compared to inhibitor-centric approaches.^6^ However, one of its current major limitations is the reliance on only a small set of amenable E3 ligases.^7^ Existing degrader modalities suggest that members of the cullin RING ligase (CRL) family are particularly compatible with TPD strategies. Approximately 250 distinct CRL ligases are encoded in the human genome, organized around one of seven cullin scaffolding proteins.^8,9^ CRLs are highly dynamically regulated and often assembled and actively decommissioned based on substrate availability.^10,11^ Both processes are dependent on the deposition and removal of the small ubiquitin-like modifier NEDD8.^12^ Attachment of NEDD8 on the cullin backbone stabilizes an active CRL complex primed for ubiquitination of a substrate, enhancing its catalytic ability up to 2000-fold.^13^ Conversely, “de-neddylation” from the cullin backbone by the COP9 signalosome (CSN)^14^ allows the exchange of substrate receptors (SRs) of CRLs, thereby reshaping the ubiquitinated proteome in a cell.^15^ In the absence of continuous substrate supply, this enables adaptation to cellular stimuli. If CRL decommissioning is perturbed, for instance, by a dysfunctional CSN, the respective CRL is hence arrested in a continuous state of activity. In the absence of substrate, this eventually leads to a process termed “auto-degradation” where the CRL-associated E2 ubiquitinates the substrate receptor.^16,17^

Only a few CRLs have thus far been harnessed for TPD, primarily via so-called hetero-bifunctional proteolysis targeting chimeras (PROTACs).^18^ These degraders bind the E3 ligase and the POI with distinct chemical moieties connected by a linker. The modular design of PROTACs allows facile chemical and thereby neo-substrate alteration. However, their degradable proteomic space is limited to ligandable targets. Molecular glue degraders (MGDs), on the other hand, are monovalent small molecules that stabilize a recognition surface between the ligase and the POI via degrader–protein and protein–protein interactions (PPIs). Mechanistic dissection of the clinically approved immunomodulatory drugs (IMiDs) has unveiled such a mechanism for the CRL4^CRBN^-dependent degradation of zinc finger transcription factors.^19−21^ Similar studies have further identified MG degraders of splicing^22,23^ and translation factors^24^ via a limited set of E3 ligases including CRL^DCAF15^.

Recent advances in chemo-proteomics workflows have augmented the identification of E3 ligase binders conducive to PROTAC development.^25−29^ However, this has not yet led to the identification of novel MGDs. Rational MGD discovery would benefit from a technology that measures drug-induced changes in the E3 ligase interactome in an unbiased fashion. Methods yielding such proteome-wide interaction data however lack the required throughput. At the same time, prospective high-throughput degrader discovery has traditionally been confined to readouts covering predefined ligase–substrate pairs using recombinant proteins.^30^

Here, we leverage the unique regulatory dynamics of CRLs to design a scalable assay informing on drug-induced changes to the interactome of a predefined ligase of interest. We find that degrader-mediated neo-substrate recruitment to a CRL rescues its SR from experimentally induced auto-degradation. Dynamic SR “tracing” via life-cell bioluminescence quantification allows degrader screening in a target agnostic, scalable, and time-resolved manner. We first benchmark this assay with known PROTACs and MGDs targeting CRL4^CRBN^ and CRL2^VHL^. Next, we determine the number of E3 ligases amendable to this approach by measuring substrate receptor auto-degradation upon CSN inhibition. Among the destabilized ligases, we select CRL^DCAF15^ for performing in-depth mechanistic studies and a proof-of-concept screen with a bespoke library of 10,000 compounds. During hit validation, the compound dRRM-1 proved to be a DCAF15-dependent, chemically distinct molecular glue degrader of RBM39 and RBM23. Taken together, the presented technology empowers the scalable identification of molecular glue degraders specific to a ligase of interest.

## Results and Discussion

### E3 Ligase Abundance Serves as a Proxy for Neo-Substrate Recruitment to E3 Ligases

**Figure 1:**
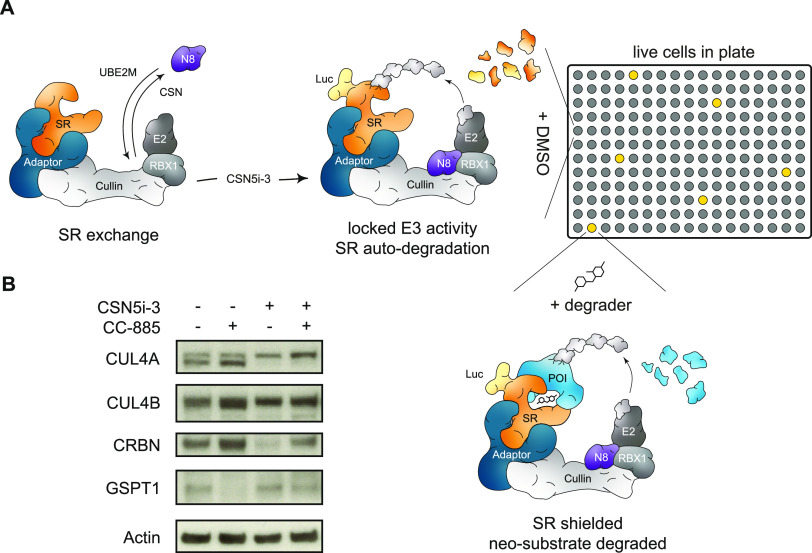
Degrader-induced neo-substrate recruitment rescues CRBN from auto-degradation. (A) Schematic depiction of the ligase tracing approach. Cullin RING ligase decommissioning is mediated through removal of NEDD8 (N8) from the cullin backbone via the COP9 Signalosome (CSN). Inhibition of the CSN locks cullin ligases in an active conformation leading to auto-ubiquitination and degradation of the luciferase-tagged substrate receptor. In 96- and 384-well plates, the addition of a degrader compound can shield the substrate receptor and rescue its auto-degradation leading to the detection of luciferase signal. (B) Protein levels in KBM7 WT cells pretreated for 10 min with DMSO or CC-885 (100 nM) followed by treatment with DMSO or CSN5i-3 (500 nM) for 4 h. Representative images of *n* = 2 experiments.

CRL activity has been implicated in degrader potency and used for the identification of novel MGDs.^31,32^ The current approaches however are limited to targets essential for cellular viability and do not allow ligase-focused discovery efforts. Active CRLs, in the absence of their substrate, can ubiquitinate their own substrate receptor in a process termed auto-degradation.^17,33,34^ This fundamental mechanism has been implicated in CRL adaptation to substrate availability and cellular stimuli.^10,11^ Here, we envisioned that chemically reestablishing substrate availability by degrader-mediated recruitment of a neo-substrate to a constitutively active, auto-degrading CRL should stabilize the ligase. Ubiquitination will be deflected from the SR to the neo-substrate and consequently increase the SR abundance (Figure 1A). To test this hypothesis, we treated the near-haploid chronic myeloid leukemia cell line HAP1 with the CRL4^CRBN^-dependent GSPT1 molecular glue degrader CC-885.^24^ Indeed, we observed that CC-885 treatment led to a subtle increase in CRBN levels, presumably via rescue of auto-degradation (Figure 1B). However, based on the minor change compared to the vehicle treatment (dimethylsulfoxide, DMSO), we surmised that steady-state CRBN auto-degradation has only a subtle contribution to its turnover (compare CRBN levels in lanes 1 and 2). Given that cullin scaffold engagement of each of the ∼250 SRs varies greatly,^10,11^ also their auto-degradation will depend on factors such as cell type and state. We therefore reasoned that enrichment of the pool of constitutively active CRLs allows the augmentation of the dynamic range of this observation (Figure 1A). NEDD8 is the central post-translational modification governing CRL activity^13^ and treatment with the de-neddylation inhibitor CSN5i-3 was previously shown to lock CRLs in a state of constitutive activation that leads to auto-degradation.^34,35^ Indeed, treatment of HAP1 cells with 500 nM CSN5i-3 yielded a significant destabilization of the CRBN substrate receptor. As hypothesized, recruitment of GSPT1 via CC-885 rescued the induced auto-degradation almost to DMSO-treated levels (Figure 1B).^24^ This striking response to molecular glue treatment led us to next explore possibilities to develop a discovery approach where novel degraders are identified by their ability to rescue a CRL from a constant state of auto-degradation. Compared to existing methods, this strategy would enable the identification of degraders specifically for an *a priori* defined ligase. Moreover, it is not restricted to POIs that are essential for cellular fitness or are otherwise implied in any measurable cellular phenotype. To make this concept amenable to degrader discovery at scale, we next sought to advance this approach toward scalable protein abundance measurement across many timepoints upon drug treatment.

### Charting CRLs Amenable to Dynamic Tracing of E3 Ligase Abundance

**Figure 2:**
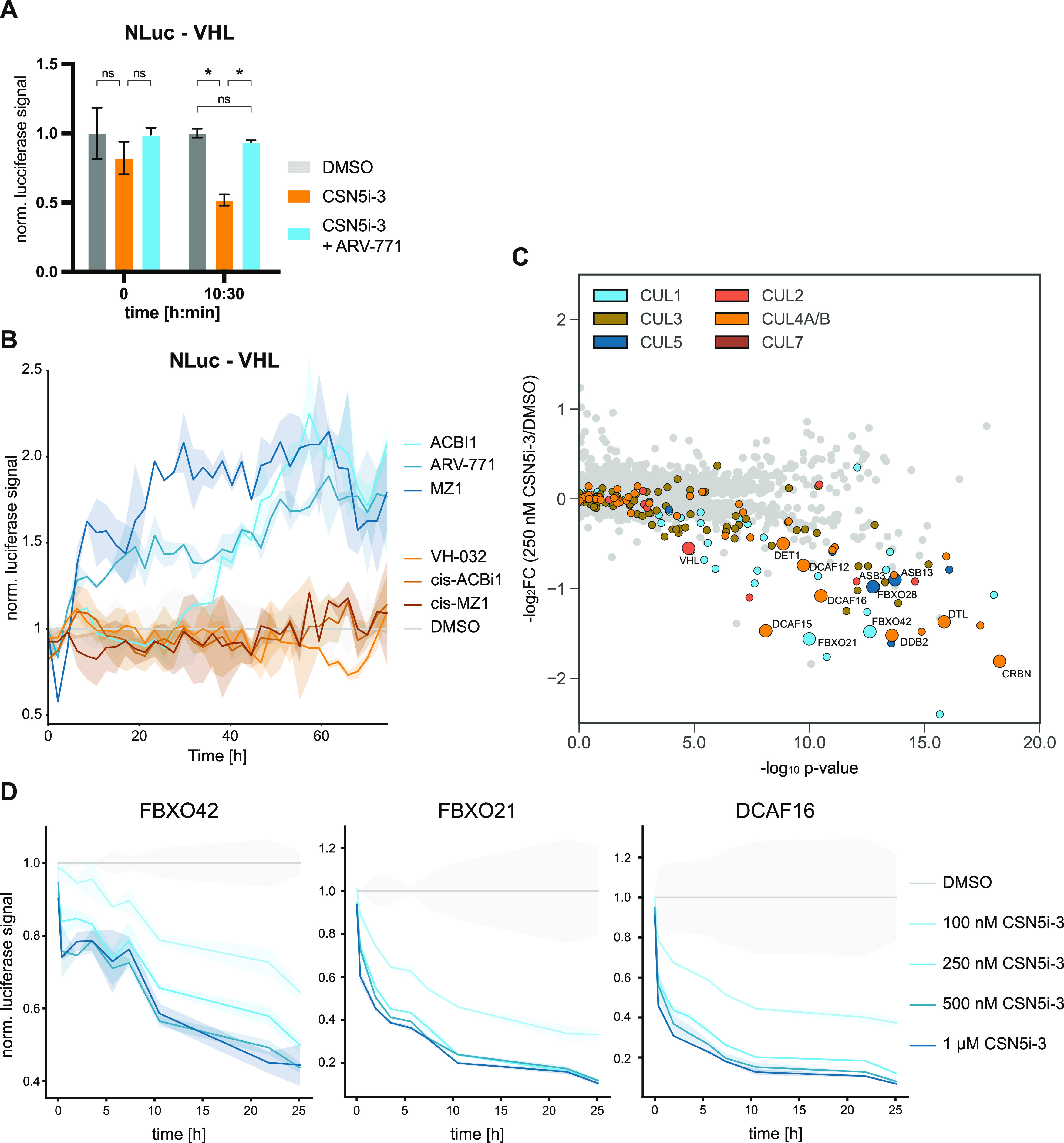
Mapping E3 ligases amendable to ligase tracing. (A) Lytic luciferase measurement of HAP1 VHL-NanoLuc knock-in cells at the indicated timepoints after treatment with DMSO, CSN5i-3 (100 nM), or CSN5i-3/ARV-771 co-treatment (100 and 500 nM, respectively). Luciferase signal is normalized to DMSO treatment at each timepoint. Mean ± standard deviation (SD) of *n* = 2 replicates. (B) Live-cell luciferase measurement of HAP1 VHL-NanoLuc expressing cells treated with CSN5i-3 (100 nM) or CSN5i-3 and the indicated degrader (100 and 500 nM, respectively). Luciferase signal is normalized to DMSO treatment at each timepoint. Mean ± SD of *n* = 2 replicates. Representative data of *n* = 2 experiments. (C) Volcano plot depicting global log_2_-fold changes of protein abundance in HAP1 cells treated with CSN5i-3 (250 nM) for 8 h. CRL substrate receptors are labeled in the indicated colors. SRs selected for validation via luciferase tagging are highlighted. Data of *n* = 3 replicates. (D) DMSO normalized live-cell luciferase signal of HAP1 cells harboring endogenous NanoLuc knock-ins for the indicated SRs. Cells were treated with DMSO or CSN5i-3 at indicated concentrations and measured over time. Mean ± SD of *n* = 2 replicates. Statistical analysis via a two-tailed *t*-test (α = 0.05), *P < 0.05.

We next proceeded to validate and benchmark the idea of “ligase tracing” for degrader identification with CRL2^VHL^, a ligase repeatedly employed for targeted protein degradation via PROTACs.^36,37^ Generating VHL-NanoLuc knock-in HAP1 cells allowed us to measure VHL abundance in lytic measurements in 384-well plate format. Upon induction of auto-degradation via CSN5i-3 treatment, VHL was destabilized within hours (Figure 2A). In live-cell measurements, we further found this to be a dose- and time-dependent process, strengthening the causal link between CRL activity and SR auto-degradation (Figure S1A). Consistent with our hypothesis, co-treatment with the BET PROTACs ARV-771 and MZ1 profoundly rescued auto-degradation of endogenous VHL (Figures 2A and S1A).^38,39^ Overexpression of NanoLuc-tagged VHL further validated these results where BET PROTACs showed dramatic VHL stabilization (data are normalized to the auto-degraded state induced by CSN5i-3 treatment, Figure 2B). Live-cell measurements also allowed us to compare responses of active BET PROTACs to inactive negative controls, and to further expand the target space to additional substrates. In line with the proposed mechanism, the enantiomer *cis*-MZ1 which is deficient for VHL binding did not elicit a response in our ligase tracing (Figure 2B). This was also validated in assays performed with NanoLuc-VHL knock-in cells (Figure S1A). Similarly, the SMARCA2/4 degrading PROTAC^40^ ACBI1 showed a pattern where only the active compound provoked changes in the auto-degradation behavior of VHL, while the enantiomer that is deficient for VHL binding failed to stabilize VHL (Figure 2B). Next, we excluded that VHL stabilization is prompted simply by small-molecule binding to VHL. Indeed, no stabilization was observed with the VHL ligand VH-032 (Figures 2B and S1A). In sum, these chemical controls validate the dependency of ligase tracing on ternary complex formation and dual-target engagement for a positive stabilization signal. Overall, this indicates that CRL substrate receptor abundance can be used as a proxy for degrader-induced neo-substrate recruitment.

CRL2^VHL^ and CRL4^CRBN^ are very well-characterized E3 ligases in TPD. To significantly expand the space of degraders and neo-substrates, we next set out to assay which CRLs are amendable to our ligase tracing assay. As the active CRL pool is shaped to the particular cellular needs at any given time, this is likely cell-line- and cell-state-specific. We chose to perform global proteomics in HAP1 cells, given that its near-haploid karyotype facilitates endogenous tagging of SRs with NanoLuc. Expression proteomics experiments were recorded after 250 nM and 1 μM CSN5i-3 treatment for 8 h (Figures 2C and S1B). In line with our hypothesis, most of the destabilized proteins were cullin-associated substrate receptors. Among these destabilized CRLs, we selected 12 SRs for either a NanoLuc knock-in or overexpression strategy in HAP1 cells. Measuring the SR abundance upon CSN5i-3-induced auto-degradation resulted in dose- and time-dependent destabilization (Figures 2D and S1C). This mimics our previous results and highlights that the ligase tracing approach can be expanded to many CRLs.

### Ligase Tracing Screen Identifies a Novel DCAF15-Dependent Molecular Glue Degrader

Discovery of novel molecular glue degraders has historically been driven by chance. After benchmarking the ligase tracing strategy with known degraders, we next set out to validate it in a chemical screening approach. To this goal, we chose to adopt the ligase tracing approach for CRL4^DCAF15^. DCAF15 can be targeted by aryl sulfonamides such as indisulam to recruit and ubiquitinate the splicing factor RBM39. Hence, it enabled us to assemble a bespoke chemical library around a chemotype known to engage DCAF15.^22,23^ Additionally, our previous results suggested that DCAF15 is strongly auto-degraded upon CSN inhibition, thus making it eligible to ligase tracing (Figure 2C).

**Figure 3:**
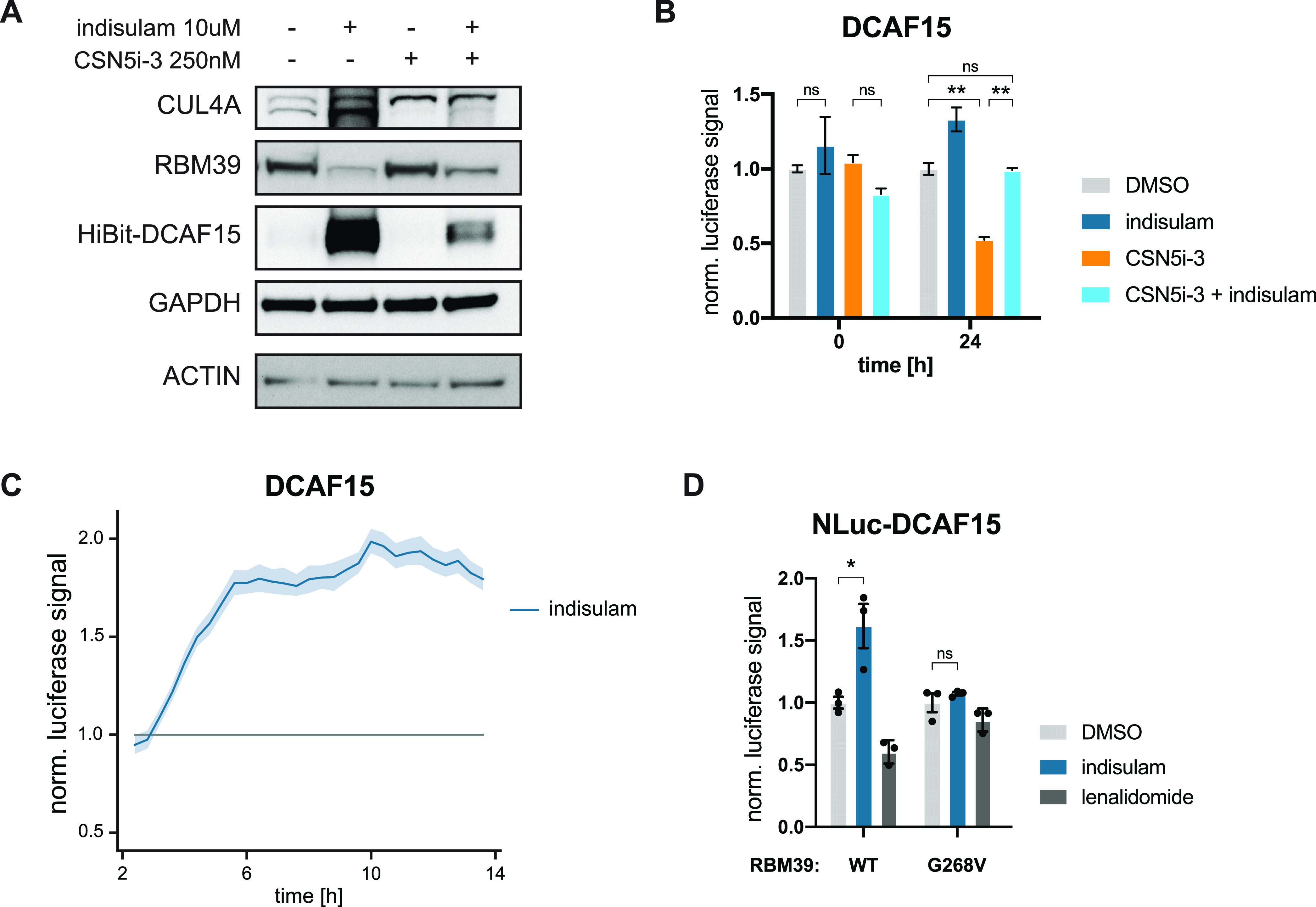
Ligase tracing detects molecular glue degrader-dependent DCAF15 stabilization. (A) Protein levels in HEK293t DCAF15^–/–^ cells with reconstitution of HiBit-DCAF15 treated with indisulam (10 μM) or CSN5i-3 (250 nM) for 24 h. Representative images of *n* = 2 experiments. (B) Bar graph depicting DMSO normalized lytic luciferase signal of HEK293t DCAF15^–/–^ cells with reconstitution of HiBit-DCAF15 + LgBit measured at the indicated timepoints after treatment with DMSO, indisulam (10 μM), CSN5i-3 (250 nM) or CSN5i-3/indisulam co-treatment (250 nM and 10 μM, respectively). Mean ± SD of *n* = 2 replicates. Representative data of *n* = 2 experiments. (C) DMSO normalized live-cell luciferase signal of HEK293t DCAF15^–/–^ cells with reconstitution of HiBit-DCAF15 + LgBit treated with indisulam (10 μM) or DMSO. Mean ± SD of *n* = 3 replicates. Representative data of *n* = 3 experiments. (D) Bar graph depicting DMSO normalized live-cell luciferase signal of HAP1 WT and RBM39^G268V^ cells with ectopic expression of HiBit-DCAF15 + LgBit measured 32 h after treatment with DMSO, indisulam (10 μM) or lenalidomide (10 μM). Mean ± SD of *n* = 3 replicates. Representative data of *n* = 2 experiments. Statistical analysis via a two-tailed *t*-test (α = 0.05), *P < 0.05, **P < 0.01.

To measure DCAF15 abundance, we initially tagged endogenous *DCAF*15 with the split luciferase 11 amino acid peptide HiBit in HEK-293 cells supplemented with the complimentary NanoLuc part LgBit.^41,42^ Upon treatment with indisulam, we observed an expected rescue of CSN5i-3-induced DCAF15 auto-degradation (Figure S2A). Due to low expression levels of endogenous *DCAF*15, we however decided to proceed with overexpression of HiBit-DCAF15 in *DCAF*15^–/–^ cells. In previous CUL4 pulldown experiments, ectopic DCAF15 expression led to a ∼21-fold increase in its cullin bound fraction.^11^ In line with such pronounced changes in assembly dynamics, we indeed encountered profound SR stabilization upon indisulam treatment even in the absence of CSN inhibition, likely due to a strong cellular auto-degradation in response to the SR overexpression (Figure 3A). This is further supported by the rescue of this auto-degradation via proteasome inhibitor treatment in these cells (Figure S2B). We next proceeded to determine DCAF15 abundance via lytic endpoint measurements. Validating our Western blot results, we observed destabilization upon CSN5i-3 treatment and profound stabilization of HiBit-DCAF15 by indisulam treatment (Figure 3B). Furthermore, we could reproduce this stabilization also in live-cell measurements and could also extend it to another previously identified RBM39 molecular glue degrader (dCeMM1) (Figures 3C and S2C).^32^ Next, we set out to test whether indisulam-mediated stabilization was specific to DCAF15 by performing ligase tracing in a variety tagged NanoLuc -SR cells. Indeed, rescue of ligase degradation was specific to HiBit-DCAF15 cells, while DCAF16, FBXO21, VHL, and FBXO42 NanoLuc knock-ins remained unchanged (Figure S2D). Importantly, the increase in DCAF15 abundance was not driven through changes in RNA expression as exemplified by *DCAF*15 quantitative polymerase chain reaction (qPCR) (Figure S2E).

In its initial identification, RBM39 degradation by indisulam was shown to be dependent on the glycine residue 268 in RBM39.^22^ Mutation of this amino acid to a valine abrogated neo-substrate recruitment and degradation.^43−45^ This critical dependency on a given protein surface topology enabled us to genetically validate that the observed DCAF15 stabilization is indeed caused by functional neo-substrate recruitment. To this end, we endogenously engineered an RBM39^G268V^ mutation in near-haploid HAP1 cells overexpressing HiBit-DCAF15 (Figure 3D). Indeed, indisulam only induced a stabilization effect in the RBM39^WT^ cells, while no change could be detected in an RBM39^G268V^ background. Of note, the CRL4^CRBN^ molecular glue degrader lenalidomide did not show any stabilization effect (Figure 3D). Together, this indicates that stabilization of DCAF15 with different RBM39 degraders is dependent on drug-induced neo-substrate recruitment. Furthermore, this highlights how CRL4^DCAF15^ presents a viable system for molecular glue degrader identification via our ligase tracing approach.

**Figure 4:**
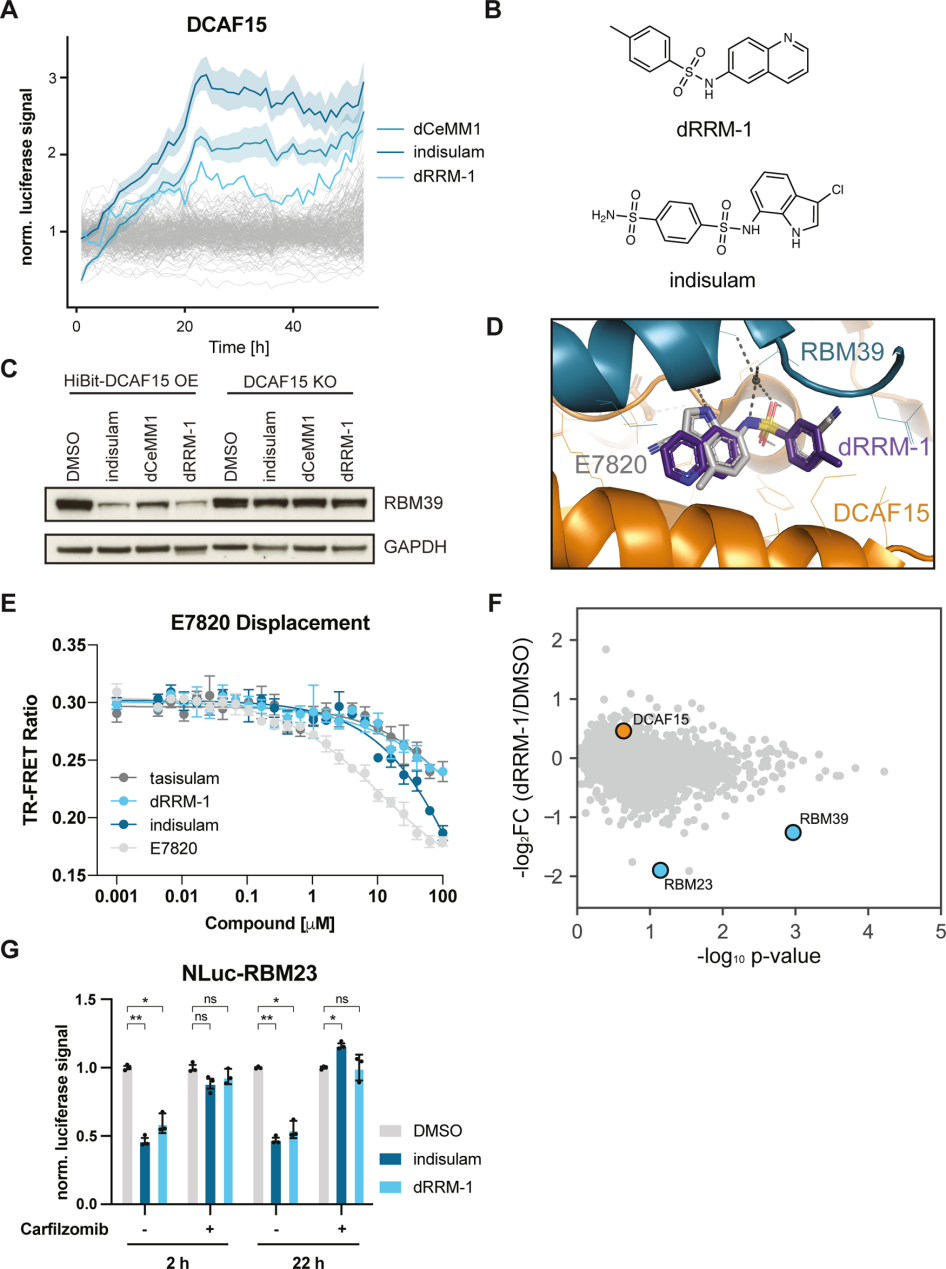
Ligase tracing screen for DCAF15 MGDs identifies dRRM-1. (A) DMSO normalized live-cell luciferase signal of HEK293t DCAF15^–/–^ cells with reconstitution of HiBit-DCAF15 + LgBit treated with 10 μM control compounds (indisulam, dCeMM1 or DMSO) or screening compounds (10 μM each, 2000 compounds shown). For indisulam and dCeMM1, data represent mean ± SD of all measured wells. (B) Chemical structures of dRRM-1 and indisulam. (C) Protein levels in HEK293t DCAF15^–/–^ cells with reconstitution of HiBit-DCAF15 treated with indisulam, dCeMM1, or dRRM-1 for 10 h. Representative images of *n* = 2 independent experiments. (D) Overlay of molecular docking of dRRM-1 in the crystal structure of DDB1ΔB-DDA1-DCAF15:E7820:RBM39 (PDB: 6Q0R). (E) TR-FRET ratio of BODIPY FL-E7820 displacement from biotinylated, terbium labeled, Strep-II-Avi-tagged DCAF15 with increasing amounts of tasisulam, dRRM-1, indisulam, or positive control E7820. The emission ratio of 520 nm (BODIPY FL) over 490 nm (terbium) is calculated and depicted as mean ± SD from *n* = 3 replicates. (F) Volcano plot depicting global log_2_-fold changes of protein abundance in HEK293t DCAF15^–/–^ cells with ectopic expression of HiBit-DCAF15 and LgBit treated with dRRM-1 (10 μM) for 10 h. Data of *n* = 2 independent measurements. (G) Bar graph depicting DMSO normalized live-cell luciferase signal of HAP1 RBM23-NanoLuc knock-in cells measured at the indicated timepoints after treatment with DMSO, indisulam (10 μM) or dRRM-1 (10 μM). Mean ± SD of *n* = 3 replicates. Statistical analysis via a two-tailed *t*-test (α = 0.05), *P < 0.001, **P < 0.0001.

Having established live-cell ligase tracing for CRL4^DCAF15^, we next set out to determine this assay’s viability for degrader discovery screening. To this end, we assembled a library of 10,000 sulfonamides of which ∼ 8000 were aryl sulfonamides leveraging the known MG chemical space for DCAF15. Ligase tracing for DCAF15 with this library showed a profound stabilization of SR levels with positive controls (indisulam, dCeMM1) in concordance with previous results (Figure 4A). As this assay harbors the inherent advantages of gain-of-signal approaches,^46^ we found a surprisingly low hit rate among other sulfonamides. Next, we selected approximately 400 compounds for a secondary validation screen (Figure S3A). Interestingly, similar stabilization effects to indisulam and dCeMM1 were only observed for the aryl sulfonamide we termed dRRM-1, which shared structural similarity to previously identified MG degraders (Figures 4A,B and S3A). Given this similarity, we next measured HiBit-RBM39 levels in knock-in cells upon treatment with these hits as well as with 84 other compounds (Figure S3B) of our validation screen. Only the positive controls as well as dRRM-1 showed clear RBM39 degradation, which in orthogonal assays proved dependent on DCAF15 (Figure 4C). Upon docking of dRRM-1 to a published crystal structure of DDB1ΔB-DDA1-DCAF15-E7820-RBM39, we further identified a shared binding mode with the previously described sulfonamide degrader E7820, suggesting a similar mode of action via CRL4^DCAF15^-mediated degradation of RBM39 (Figure 4D).^43−45^ Similarly to known RBM39 degraders such as tasisulam, measurement of E7820 displacement from DCAF15 by dRRM-1 in a TR-FRET assay revealed a comparatively low binding affinity to DCAF15 (Figure 4E).^45^ This however highlights how ligase tracing can detect functionally relevant glue degraders, even though they have comparatively low ligase affinity. Comparing dRRM-1 and indisulam-mediated toxicity and RBM39 degradation in different cellular backgrounds supported this lower DCAF15 engagement (Figure S3C,D). Global proteomics experiments revealed that not only RBM39 is degraded via dRRM-1 treatment but also the closely related splicing factor RBM23 (Figure 4F). RBM23 shares a high sequence similarity to RBM39 and has previously been shown to be targeted by other sulfonamides.^45,47^ Intrigued by the potential degradation of RBM23 over RBM39 by dRRM-1, we generated a C-terminal RBM23-NanoLuc knock-in HAP1 cell line and measured its abundance upon sulfonamide treatment. Indisulam and dRRM-1 led to similar time-dependent RBM23 degradation, which could be rescued by co-treatment with the proteasome inhibitor carfilzomib (Figure 4G).

In summary, we outline and validate a CRL-centric phenotypic screening approach that allowed us to identify dRRM-1, a DCAF15-dependent MG degrader which retains a binding mode similar to previously described, and serendipitously identified degraders.

## Conclusions

TPD promises a paradigm shift in drug discovery by overcoming the limitations of inhibitor-centric, occupancy-driven pharmacology through its catalytic components. In addition to expanding the druggable proteome, areas of interest include tissue- and cell-state selective degradation of disease-relevant proteins. Delivering these promises is currently severely limited as only 2% of E3 ligases are amenable to TPD. Methods based on chemo-proteomics have led to the discovery of covalent E3 ligase binders, which in turn have spurred PROTAC development.^25−29^

Here, we outlined a strategy of measuring drug-induced changes to the interactome of an E3 ligase of choice by leveraging the regulatory circuits of cullin RING ligases. We benchmark this scalable assay with the two best-studied E3 ligases in TPD, CRL4^CRBN^ and CRL2^VHL^. By use of the CRL2^VHL^ binding ligand VH-032, we show that drug-ligase engagement is insufficient to rescue a ligase from auto-degradation. Instead, the ligase tracing assay specifically reports on drug-induced neo-substrate recruitment. We further profile all E3 ligases amendable to this approach in our given cell line model and choose to perform in-depth studies with CRL4^DCAF15^ given its pronounced auto-degradation and the availability of aryl sulfonamides as a known chemotype potentially capable of co-opting DCAF15. A single point mutation abrogating MGD-dependent recruitment of RBM39 to CRL^DCAF15^ was sufficient to disrupt ligase tracing signal highlighting the assay specificity. Among 10,000 sulfonamides tested, we identified dRRM-1 a molecular glue degrader of RBM39 and RBM23 and validate its mode of action via TR-FRET and global proteomics. We conclude that our ligase tracing assay allows identification of functional degrader molecules in an E3 ligase selective but target agnostic way.

This allows selection of therapeutically enticing CRL E3 ligases, taking into account their characteristics such as disease relevance, expression pattern, and target complementarity. In fact, recently, we have shown that essentiality of an E3 ligase can have a profound impact on the emergence of resistance to degrader modalities, further highlighting the need to expand the targetable E3 ligase space.^48^ In principle, ligase tracing assays capture POI recruitment on a proteome-wide scale. Future research will be required to determine the threshold of neo-substrate abundance required for this assay. Of note, the explored neo-substrate space can likely be biased by overexpressing pools of targets of interest. An advantage of ligase tracing over other previously reported methods for molecular glue discovery lies in its independence from the neo-substrate’s essentiality status. While discovery of cyclin K molecular glue degraders hinged on their cytotoxicity, the here presented method directly reports on changes to the interactome of an E3 ligase. The general gain-of-signal design of ligase tracing also harbors advantages such as increasing rates of true positives. Moreover, it is conceivable that drug-indued recruitment of different neo-substrates might prompt different kinetics of ligase re-stabilization, based on the steric considerations and abundance of a given POI. While future research will be needed to unequivocally validate this hypothesis, ligase tracing of VHL has indicated differentiated stabilization curves for BET PROTACs compared to the SMARCA2/4 PROTAC ACBI1. Finally, one can envision a multiplexing strategy by measuring the abundance of several E3 ligases in the same well via fluorescent protein tagging coupled to a microscopy.

Of note, a gain of signal in ligase tracing does not necessarily require neo-substrate degradation but could also be caused by disassembly of the specific ligase, by inhibition of the associated E2, or by even more pleiotropic UPS perturbations. However, such false positives would be identified for most assayed ligases and can hence easily be eliminated. Overall, we believe that the outlined method can be easily adopted to other E3 ligases of interest and facilitate the *de novo* identification of E3 ligase binders and molecular glue degraders in a target agnostic fashion.

## Supporting Information Available

Materials and Methods (Figures S1–S3) (PDF)
Primers and plasmids (Table S1) (XLSX)
Proteomics results and chemical library information (Table S2) (XLSX)
