# Supplementary material for: E3-Specific Degrader Discovery by Dynamic Tracing of Substrate Receptor Abundance
Source: J Am Chem Soc. 2023 Jan 5;145(2):1176–84. doi: 10.1021/jacs.2c10784 (PMC9853857; doi:10.1021/jacs.2c10784)
Supplement: Supplementary file 1 — ja2c10784_si_001.pdf [file ja2c10784_si_001.pdf]

Supporting Information to:

E3-specific degrader discovery by dynamic tracing of substrate receptor abundance

Alexander Hanzl[1], Eleonora Barone[1,*], Sophie Bauer[1,4,*], Hong Yue[2,3], Radosław P. Nowak[2,3], Elisa Hahn[1], Eugenia V. Pankevich[1], Anna Koren[1], Stefan Kubicek[1], Eric S. Fischer[2,3], Georg E. Winter[1]

Affiliations

[1] CeMM Research Center for Molecular Medicine of the Austrian Academy of Sciences, 1090 Vienna, Austria

[2] Department of Cancer Biology, Dana-Farber Cancer Institute, Boston, MA 02215, USA

[3] Department of Biological Chemistry and Molecular Pharmacology, Harvard Medical School, Boston, MA 02115, USA

[4] present address: Proxygen GmbH, 1030 Vienna, Austria

* these authors contributed equally

[#] correspondence to gwinter@cemm.oeaw.ac.at

Supplementary Figures

Supplementary Figure 1.

(A) DMSO normalized live-cell luciferase signal of HAP1 VHL-NanoLuc knock-in cells treated with different doses of CSN5i-3 or DMSO (left). On the right, same cells co-treated with CSN5i-3 (250 nM) and ARV-771, MZ-1, VH-032, cis-MZ1 or DMSO (1 µM each). Mean of *n* = 3 replicates.

(B) Volcano plot depicting global log_2-fold changes of protein abundance in HAP1 cells treated with CSN5i-3 (1 µM) for 8 h. CRL substrate receptors are labeled in the indicated colors. SRs selected for validation via luciferase tagging are highlighted. Data of *n* = 3 replicates.

(C) DMSO normalized live-cell luciferase signal of HAP1 cells overexpressing the indicated protein in C-terminal fusion with NanoLuc. Cells were treated with DMSO or CSN5i-03 (500 nM) and measured at the indicated timepoint after treatment. Mean of n = 2 independent measurements. Representative data of *n* = 2 experiments.

Supplementary Figure 1

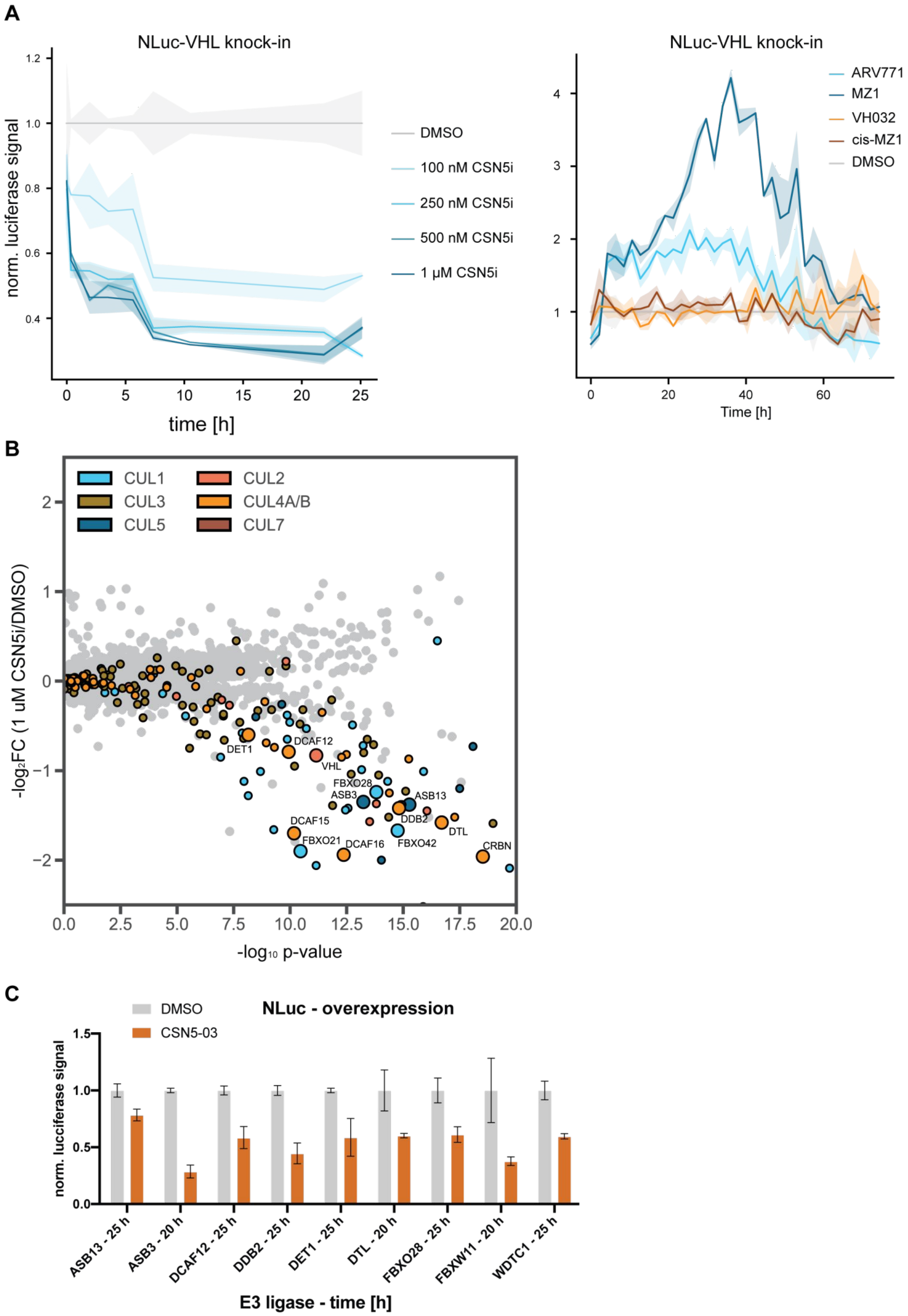

Supplementary Figure 2

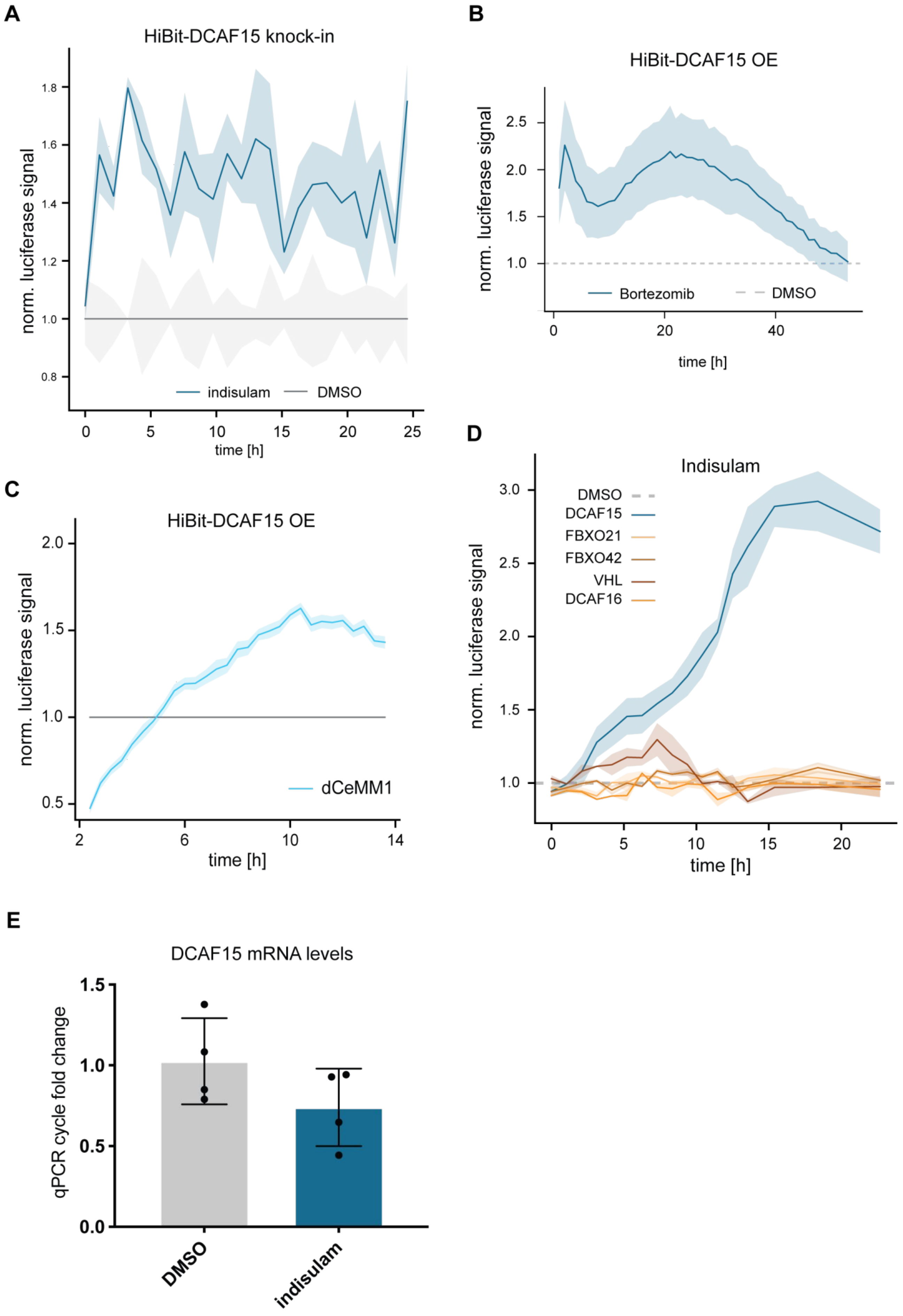

Supplementary Figure 2.

(A) DMSO normalized live-cell luciferase signal of HEK293t HiBit-DCAF15 endogenous knock-in cells with ectopic expression of LgBit treated with indisulam or DMSO (10 µM). Mean of *n* = 3 replicates.

(B and C) DMSO normalized live-cell luciferase signal of HEK293t DCAF15$^{-/-}$ cells with reconstitution of HiBit-DCAF15 + LgBit treated with 1 µM bortezomib (B) or 10 µM dCeMM1 (C) or DMSO. Mean of *n* = 3 replicates. Representative data of *n* = 2 experiments.

(D) DMSO normalized live-cell luciferase signal of HAP1 cells with endogenous knock-in of NanoLuc for the indicated proteins. Cells were co-treated with CSN5i-3 and DMSO or indisulam (250 nM and 1 µM, right) and measured at the indicated timepoints after treatment. Mean of *n* = 2 replicates. Representative data of *n* = 2 experiments.

(E) Bar graph depicting fold-change in qPCR cycles of exponential amplification in HEK293t DCAF15$^{-/-}$ cells with reconstitution of HiBit-DCAF15 + LgBit after treatment with indisulam or DMSO for 10 h. Mean of *n* = 4 replicates.

Supplementary Figure 3.

(A) DMSO normalized live-cell luciferase signal of HEK293t DCAF15$^{-/-}$ cells with reconstitution of HiBit-DCAF15 + LgBit treated with 10 µM dCeMM1, DMSO or 395 screening compounds (10 µM each). Orange compounds were selected for subsequent RBM39-HiBit degradation assay in (B)

(B) DMSO normalized live-cell luciferase signal of HCT116 RBM39-HiBit knock-in cells with ectopic expression of LgBit treated with indisulam, dCeMM1, dRRM-1, DMSO or 85 screening compounds (10 µM each). Data represents mean ± s.d. of all measured wells (2 for screening compounds, 8 wells for controls).

(C) Dose-resolved, normalized viability after 3 d treatment with dRRM-1 and indisulam in the respective cells. Mean ± s.e.m.; n = 3 independent treatments.

(D) Protein levels in the respective cells with 20 µM treatment (left) or 5 and 10 µM treatment (right) with dRRM-1 or indisulam for 10 h.

Supplementary Figure 3

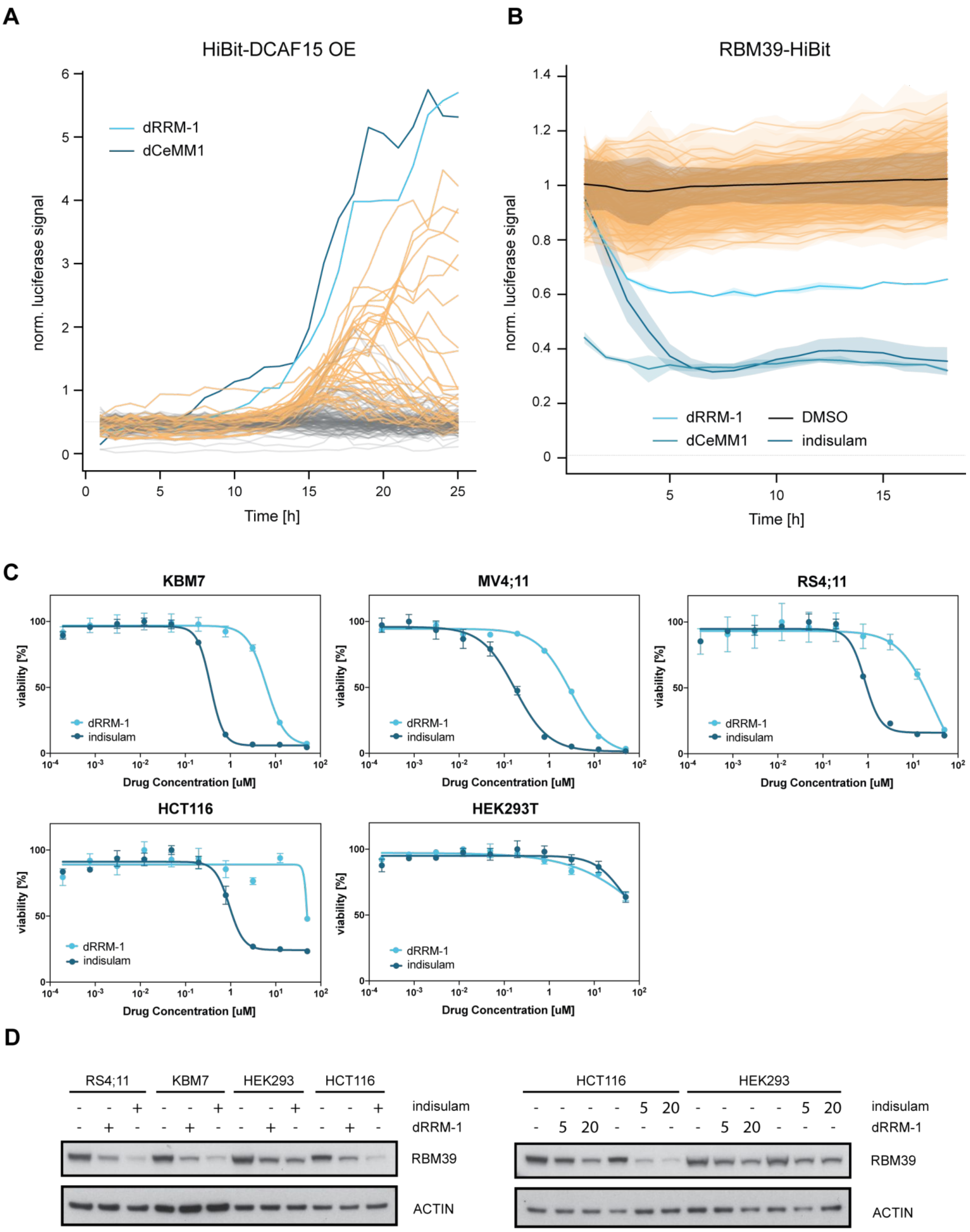

Materials and Methods

Cell lines, tissue culture and lentivirus production

KBM7 cells (a gift from T. Brummelkamp) and HAP1 cells were grown in IMDM supplemented with 10% Fetal Bovine Serum and 1% penicillin/streptomycin (100 units/ml Penicillin, 100 µg/ml Streptomycin; pen/strep). HEK293 cells (a gift by the Bradner Lab) and HCT116 cells (a gift by the Superti-Furga Lab) were grown in in DMEM supplemented with 10% Fetal Bovine Serum and 1% pen/strep. MV4;11 and RS4;11 cells were cultured in RPMI supplemented with 10% Fetal Bovine Serum and 1% pen/strep. pSpCas9(BB)-2A-GFP (PX458) or pSpCas9(BB)-2A-Puro (PX459) was obtained through Addgene (48138 and 62988) and used to transiently express sgRNA against CRBN, VHL, DCAF15 and other genes for knock-out and knock-in generation (sgRNA sequences are summarized in Supplementary Table 1). Clones were single cell seeded and checked for gene deletion via PCR on gDNA or Western blotting. For lentiviral production, 293T cells were seeded in 10 cm dishes and transfected at approx. 80 % confluency with 4 µg target vector, 2 µg pMD2.G (Addgene 12259) and 1 µg psPAX2 (Addgene 12260) using PEI and following standard protocol. The viral supernatant was harvested 72 h after transfection and filtered with a 0.45-µm syringe filter to remove cell debris. Lentivirus was then aliquoted and stored at – 80 °C until transduction of 1 x 10^6 cells in 1 ml of media plus virus in 24 well plates with the addition of 8 µg per ml polybrene (Sigma) and spin inoculation for 1 h at 2,000 r.p.m. Antibiotic selection was performed 24 to 48 h after transduction with 10 µg ml^{-1} blasticidin or 1 µg ml^{-1} puromycin.

Plasmids and cloning

All plasmids used in this study are summarized in Supplementary Table 1. For ectopic expression of E3 ligases tagged with full length NanoLuc, cDNA of the specific genes was ordered in pENTR223 vectors from the BCCM/Belspo consortium as part of the human ORFome library[49]. E3 ligase cDNAs were then cloned into pLenti6.2-ccdB-Nanoluc (Addgene 87075) via gateway LR-recombination cloning (Invitrogen) following manufacturers recommendations.

For cloning of sgRNA cutting plasmids to generate endogenous NanoLuc and HiBit knock-ins, we utilized a universal pX330A_sgX_sgPITCh cutting plasmid via adaptation of a published protocol[50]. sgRNAs targeting the endogenous locus (Supplementary Table 1) were selected to lie as close as possible to the start- or stop codon with the minimal predicted off-target activity. They were introduced to the vector via oligonucleotide annealing and subsequent BbsI-mediated restriction cloning. The second part of this micro-homology mediated knock-in strategy was introduced by adapting the pCRIS-PITChv2 repair template plasmid to contain a N-terminal blasticidin-P2A-2xHA- NanoLuc cassette which was generated via a geneblock and PCR of the flanking PITCh sgRNA target sites. This PCR product was then introduced in MluI linearized pCRIS-PITChv2 vector via NEBuilder 2× HiFi assembly (New England Biolabs). Primers containing 20 to 22 bp homology regions corresponding to the genomic locus 5' and 3' of the sgRNA cleavage were used to PCR this cassette. The resulting repair template introducing a blasticidin marker, and a double HA tagged NanoLuc to the genomic locus was reintroduced into MluI linearized pCRIS-PITChv2 vectro backbone with NEBuilder 2× HiFi assembly (New England Biolabs)[51].

Endogenous genome editing for knock-in and mutant generation

To generate cell lines expressing HiBit or NanoLuc tagged POIs, HAP1 WT or HCT116 WT cells were seeded into 6-well plates to obtain approximately 70 % confluency the next day. For microhomology mediated knock-in, in each well, PITCh sgRNA/Cas9 and repair template plasmids were transfected at 1.5 µg each via PEI following standard protocol (see Supplementary Table 1 for sgRNA and microhomology sequences). The next day, each condition was split to a 10 cm dish and antibiotic selection for successful editing was started 48 hours after transfection. Single cell selection was

ensured by limited dilution into 384 well-plates (seeding at 0.2–1 cells per well in 50 µl) or by picking single colonies directly of the plate. Successful knock-in was characterized via immunoblotting for the introduced HA-tag and/or via genotyping by PCR of the targeted genomic region.

For knock-in of the shorter HiBit-tag in the C-terminus of RBM39 in HCT116, a similar approach was used only that the repair cassette could be introduced via annealed oligos instead of an entire plasmid. For this oligos harboring the 33 bp HiBit-tag flanked by 20 bp homologies from the genomic sgRNA cut site were ordered and co-transfected as described above. Cells were pulse-selected from 24 h post transfection to 72 hrs post transfection for Cas9 expression and subsequently single cell seeded at 0.2–1 cells per well in a 384 well plate. Single clones were pre-selected by lytic Nanoluc reconstitution under treatment with indisulam or DMSO. Clones that showed loss of luciferase signal under indisulam were subsequently characterized via immunoblotting for the introduced HiBit-tag and via genotyping by PCR of the targeted genomic region.

For generation of the RBM39 G268V mutant HCT116 cells, again annealed oligos were utilized in a similar fashion. After CRISPR/Cas9 mediated cutting at the specific genomic locus a repair template harboring a 40 bp homology and the desired point mutation in its center was used to introduce the mutation. Cells were seeded and transfected with the sgRNA/Cas9 plasmid and the repair oligos as mentioned above followed by pulse-selection for Cas9 expression from 24 h post transfection to 72 hrs post transfection and subsequently single cell seeding. Next, clones were mirror-plated in 96 well plates after initial expansion and pre-selected by treatment with indisulam in one of the mirror plates. Single clones that showed resistance to indisulam were subsequently characterized via genotyping by PCR of the targeted genomic region to identify the specific introduced point mutation.

Transcript quantification via qPCR

1 x 10^6 HEK293T cells with ectopic expression of HiBit-DCAF15 and LgBit were treated with DMSO or 10 µM indisulam for 12 h, detached and RNA was isolated using the RNeasy Kit and QIAshredder (Qiagen) following standard protocol with DNA digestion. Reverse transcription PCR was performed with the RevertAID First Strand cDNA synthesis kit and Oligo-dT primers (Thermo Scientific). DCAF15 RNA was quantified in a PCR reaction using SYBR select master mix (Fisher Scientific) in the following reaction: 3.75 µl of 1:10 diluted cDNA, 0.75 µl of DCAF15_exon9-11 primer mix (10 µM each, see Supplementary Table 1), 3 µl H_2O and 7.5 µl SYBR master mix. The reaction mixture was denatured for 3' at 95 °C followed by 45 cycles of 15" at 95 °C, 45" at 60 °C and 15" at 95 °C with a final extension of 1' at 60 °C and 15" at 95 °C in a StepOne Plus real-time PCR cycler (Applied Biosystems). The cycle number for exponential amplification was determined and normalized to DMSO treated samples and visualized with Prism (GraphPad).

Western blot analysis

PBS-washed cell pellets were lysed in RIPA Buffer (50 mM Tris-HCl pH 8.0, 150 mM NaCl, 1% Triton X-100, 0.5% sodium deoxycholate, 0.1% SDS, 1× Halt protease inhibitor cocktail, 25 U ml^{-1} Benzonase). Lysates were cleared by centrifugation for 15 min at 4 °C and 20,000*g*. Protein concentration was measured by BCA according to the manufacturer's protocol (Thermo Scientific™ Pierce™ BCA Protein Assay Kit) and 4X LDS sample buffer was added. Proteins (20 µg) were separated on 4-12% SDS-PAGE gels and transferred to nitrocellulose membranes. Membranes were blocked with 5 % milk in TBST for 30 min at RT. Primary antibodies were incubated in milk or TBST alone for 1 h at RT or 4 °C overnight. Secondary antibodies were incubated for 1 h at RT. Blots were developed with chemiluminescence films. Primary antibodies used: GSPT1 (1:1000, Abcam, ab49878), CRBN (1:2000, kind gift of R. Eichner and F. Bassermann), VHL (1:1000, Cell Signaling Technology, 2738), ACTIN (1:5000, Sigma-Aldrich, A5441-.2ML), GAPDH (1:1000, Santa Cruz Biotechnology, sc-365062), RBM39 (1:2500, Sigma-Aldrich, HPA001591), CUL4A (1:1000, Cell Signaling Technology, 2699), CUL4B (1:1000, proteintech, 12916-1-AP). Secondary antibodies used:

Peroxidase-conjugated AffiniPure Goat Anti-Rabbit IgG (1:10000, Jackson ImmunoResearch, 111-035-003) and Peroxidase-conjugated AffiniPure Goat Anti-Mouse IgG (1:10000, Jackson ImmunoResearch, 115-035-003). For HiBit-tagged protein detection LgBit complementation and luminescence measurements were performed on the full membrane before blocking with 5 % milk following manufacturer's guidelines with the Nano-Glo HiBit Blotting system (Promega).

E3 ligase luciferase measurements

Live cell measurements

For NanoLuc measurements, cells were diluted to 1 M cells ml^{-1} in media and 10 µl of this suspension (10k cells) seeded in a black 384-well plate (Corning, 3764). For large scale chemical screens, compounds were dispensed with an Echo 550 system and resuspended in 10 µl of media prior to cell seeding, ensuring compound concentrations of 10 µM in the final assay volume of 40 µl. For small scale luciferase measurements, 10 µl of pre-made compound solution in media was added to each well. Positive (depending on assayed ligase) and negative (DMSO) control compounds were scattered over each plate to judge and eliminate plate positional effects. Finally, 20 µl of media supplemented with 50 mM HEPES (Sigma), 1:100 Endurazine Luciferase live cell substrate (Promega) and depending on condition, CSN5i-3 (MedChemExpress) were added to each well. Luciferase measurements were performed every 1 to 2 hours on an EnVision plate reader (PerkinElmer). Results were analysed by employing python (v3.8.5), pandas (v1.1.3) and numpy (v1.19.2) to normalize each well and timepoint per plate to its relative negative control measurement (DMSO) and depicted using matplotlib (v3.3.2) and seaborn (v0.11.0). The full chemical library information is reported in Supplementary Table 2. On average, compounds had a molecular weight of 414 g/mol, contained 3 ring structures and 8 heteroatoms.

Lytic measurements

For Lytic endpoint measurements cells were seeded as mentioned above and NanoLuc abundance was determined via the Nano-Glo HiBit lytic detection kit (Promega) following manufacturers recommendations. Depending on the cell line used (HiBit- or NanoLuc tagged protein), LgBit was added to the final measurement mix or not. Results were analysed as described above and visualized with Prism (GraphPad).

Time-resolved Förster resonance energy transfer

Protein constructs, expression and purification were performed as previously described[45]. Titrations of compounds in BODIPY FL-E7820 displacement assay were carried out by mixing 200 nM biotinylated Strep-II-Avi-tagged DCAF15 variants, 2 nM terbium-coupled streptavidin in assay buffer containing 50 mM TRIS, pH 7.5, 200 mM NaCl, 0.1% Pluronic F-68 solution (Sigma) and 5 µM of BODIPY FL-E7820. After dispensing the assay mixture, an increasing concentration of small molecules was dispensed in the 384-well plate (Corning, 4514) using a D300e Digital Dispenser (HP) normalized to 2% DMSO and then incubated for 60 min at room temperature. After excitation of terbium fluorescence at 337 nm, emission at 490 nm (terbium) and 520 nm (BODIPY FL) were recorded with a 70 µs delay over 600 µs to reduce background fluorescence, and the reaction was followed over 10 cycles of each data point using a PHERAstar FSX microplate reader (BMG Labtech). The TR-FRET signal of each data point was extracted by calculating the 520/490 nm ratio. The IC_{50} values were estimated using the variable slope equation in Prism (GraphPad). All TR-FRET results are plotted as mean ± s.d. from three independent replicates (n = 3).

Molecular Docking Analysis

The crystal structures of DCAF15-DDB1ΔB-DDA1 complex (PDB: 6Q0R) were prepared using the Protein Preparation Wizard in Maestro (Maestro release 2022-1, Epik version 5.9137). Default settings were used, except that all

crystallographic water molecules > 5 Å from heteroatom groups were removed. The docking receptor grid was created using the Receptor Grid Generation module in Glide (Glide version 94137). The grid box and center were set to default by using the active site ligand (E7820), with the active site ligand excluded from the grid. The ligands were prepared using the LigPrep module with OPLS3 force field and default settings (LigPrep version 61137). The docking poses were generated using the LigandDocking protocol as implemented in Schrödinger Suite 2022-1. Default settings were used with the Standard Precision (SP) score function with flexible ligand sampling. Briefly, the grid box and center were set at default using the active site ligand, and no constraints were defined. The top pose with the lowest Glide SP score is shown for dCeMM5. Figures were generated in PyMOL (2.5.1, Schrödinger, LLC).

Expression proteomics

Global proteomics were essentially performed as previously described.[34,35] First, we compared overall proteome-wide changes in HAP1 WT cells treated with DMSO or CSN5i-3 (1mM and 250 nM, 8h). Second, we profiled dRRM-1 treatment (10 µM for 10 hrs) in HAP1 DCAF15$^{-/-}$ cells overexpressing HiBit-DCAF15 and LgBit.

Sample preparation

30x10^6 HAP1 cells per condition were collected, washed four times with ice-cold PBS, the supernatant aspirated and pellets snapfrozen in liquid N2. Each washed cell pellet was lysed separately in 40 µL of freshly prepared lysis buffer containing 50 mM HEPES (pH 8.0), 2% SDS, 0.1 M DTT, 1 mM PMSF, and protease inhibitor cocktail (Sigma-Aldrich). Samples rested at RT for 20 minutes before heating to 99 °C for 5 min. After cooling down to RT, DNA was sheared by sonication using a Covaris S2 high performance ultrasonicator. Cell debris was removed by centrifugation at 20.000 g for 15 min at 20 °C. Supernatent was transferred to fresh eppendorf tubes and protein concentration determined using the BCA protein assay kit (Pierce Biotechnology). FASP was performed using a 30 kDa molecular weight cutoff filter (VIVACON 500; Sartorius Stedim Biotech) according to published procedures.[52] In brief, 100 ug total protein per sample were reduced by adding DTT at a final concentration of 83.3 mM followed by incubation at 99 °C for 5 min. After cooling to room temperature, samples were mixed with 200 µL of freshly prepared 8 M urea in 100 mM Tris-HCl (pH 8.5) (UA-solution) in the filter unit and centrifuged at 14.000 g for 15 min at 20 °C to remove SDS. Any residual SDS was washed out with 200 µL of UA. The proteins were alkylated with 100 µL of 50 mM iodoacetamide in the dark for 30 min at RT. Next, three washing steps with 100 µL of UA solution were performed, followed by three washing steps with 100 µL of 50 mM TEAB buffer (Sigma-Aldrich). Proteins were digested with trypsin at a ratio of 1:50 overnight at 37 °C. Peptides were recovered using 40 µL of 50 mM TEAB buffer followed by 50 µL of 0.5 M NaCl (Sigma-Aldrich). Peptides were desalted using C18 solid phase extraction spin columns (The Nest Group). After desalting, peptides were labeled with TMT 10-plex reagents according to the manufacturer protocol (Pierce Biotechnology). After quenching of the labeling reaction, labeled peptides were pooled, organic solvent removed in vacuum concentrator and labeled peptides cleaned via C18 solid phase extraction.

Offline Fractionation via RP-HPLC at high pH

Tryptic peptides were re-buffered in 20 mM ammonium formiate buffer pH 10, shortly before separation by reversed phase liquid chromatography at pH 10 as described[53]. Peptides were separated into 96 time-based fractions on a column (150 x 2.0 mm Gemini-NX 3 µM C18 110 Å, Phenomenex) using an Agilent 1200 series HPLC system fitted with a binary pump delivering solvent at 100 uL/min. Acidified fractions were consolidated into 36 fractions via a concatenated strategy described[54]. After solvent removal in a vacuum concentrator, samples were reconstituted in 5% formic acid for LC-MS/MS analysis and kept at -80 °C until analysis.

2D-RP/RP Liquid Chromatography Mass Spectrometry

Mass spectrometry was performed on an Orbitrap Fusion Lumos mass spectrometer (ThermoFisher Scientific) coupled to a Dionex Ultimate 3000RSLC nano system (ThermoFisher Scientific) via nanoflex source interface. Tryptic peptides

were loaded onto a trap column (Pepmap 100, 5 μM, 5 x 0.3 mm, ThermoFisher Scientific) at a flow rate of 10 uL/min using 2% ACN and 0.05% TFA as loading buffer. After loading, the trap column was switched in-line with a 40 cm, 75 μM inner diameter analytical column (packed in-house with ReproSil-Pur 120 C18-AQ, 3 μM, Dr. Maisch). Mobile-phase A consisted of 0.4% formic acid in water and mobile-phase B of 0.4% formic acid in a mix of 90% acetonitrile and 9.6% water. The flow rate was set to 230 nL/min and a three-step 90 min gradient applied (6 to 30% solvent B within 81 min, 30 to 65% solvent B within 8 min, and 65 to 100% solvent B within 1 min, 100% solvent B for 6 min before equilibrating at 6% solvent B for 18 min prior to next injection). Analysis on the MS was performed in a data-dependent acquisition (DDA) mode using a maximum 3 s cycle time. Full MS^1 scans were acquired in the Orbitrap with a scan range of 375 - 1650 m/z and a resolution of 120,000 (at 200 m/z). Automatic gain control (AGC) was set to a target of 2×10^5 and a maximum injection time of 50 ms. MS^2 spectra were acquired in the Orbitrap at a resolution of 50,000 (at 200 m/z) with a fixed first mass of 100 m/z. A tandem MS approach was chosen (TMT reporter ion intensities extracted from MS^2 scans) to achieve maximum proteome coverage. To minimize TMT ratio compression effects by interference of contaminating co-eluting isobaric peptide ion species, precursor isolation width in the quadrupole was set to 0.4 Da and an extended fractionation scheme applied (36 fractions, see above). Monoisotopic peak determination was set to peptides with inclusion of charge states between 2 and 7. Intensity threshold for MS^2 selection was set to 5×10^4. Higher energy collision induced dissociation (HCD) was applied with a normalized collision energy (NCE) of 38%. AGC was set to 1×10^5 with a maximum injection time of 105 ms. Dynamic exclusion for selected ions was 60 s. A single lock mass at m/z 445.120024 was employed. Xcalibur (v4.2.28.14) and Tune (v3.1, 2412.17) were used to operate the instrument.

Data Analysis

Acquired raw data files were processed using Proteome Discoverer (v2.2.0) with the Sequest HT database search engine and Percolator validation software node (v3.04) to remove false positives with a false discovery rate (FDR) of 1% on peptide and protein level under strict conditions. Searches were performed with full tryptic digestion against the human SwissProt database v2017.06 with up to two allowed miscleavage sites. Oxidation (+15.9949Da) of methionine was set as variable modification, while carbamidomethylation (+57.0214Da) of cysteine residues and TMT labeling of peptide N-termini and lysine residues were set as fixed modifications. Data was searched with mass tolerances of ± 10 ppm and ± 0.02Da on the precursor and fragment ions, respectively. Results were filtered to include peptide spectrum matches (PSMs) with Sequest HT cross-correlation factor (Xcorr) scores of ≥ 1 and high peptide confidence assigned by Percolator. MS^2 signal-to-noise values (S/N) values of TMT reporter ions were used to estimate peptide/protein abundance changes. PSMs with precursor isolation interference values of ≥ 50% and average TMT-reporter ion S/N ≤ 10 were excluded from quantitation. Only unique peptides were used for TMT quantitation. Isotopic impurity correction and TMT channel normalization based on total peptide amount were applied. For statistical analysis *and P*-value calculation, the integrated ANOVA test was used. TMT ratios with *P*-values below 0.01 were considered as significant. Only proteins with > 1 peptide detected and > 1 unique peptide detected were considered for further analysis. A volcano plot was generated for all proteins detected by depicting log2 fold changes in TMT-ratios for CSN5i treatment vs. DMSO against the protein specific *P*-values. For visualizing dRRM-1 destabilized proteins a similar plot was generated with changes between degrader treatment and DMSO. Full results of global proteome changes can be found in Supplementary Table 2.

Data availability

All data presented in this study is available in main figures, supplementary information or upon request from the authors. Full proteomics results and chemical library information are available in the supplementary information.

References

49. Yang, X. et al. A public genome-scale lentiviral expression library of human ORFs. *Nat Methods* **8**, 659-661 (2011).
50. Jaeger, M. G. et al. Selective Mediator dependence of cell-type-specifying transcription. *Nat Genet* **52**, 719-727 (2020).
51. Brand, M. & Winter, G. E. Locus-Specific Knock-In of a Degradable Tag for Target Validation Studies. *Methods Mol Biol* **1953**, 105-119 (2019).
52. Wiśniewski, J. R., Zougman, A., Nagaraj, N. & Mann, M. Universal sample preparation method for proteome analysis. *Nat Methods* **6**, 359-362 (2009).
53. Gilar, M., Olivova, P., Daly, A. E. & Gebler, J. C. Two-dimensional separation of peptides using RP-RP-HPLC system with different pH in first and second separation dimensions. *J Sep Sci* **28**, 1694-1703 (2005).
54. Wang, Y. et al. Reversed-phase chromatography with multiple fraction concatenation strategy for proteome profiling of human MCF10A cells. *Proteomics* **11**, 2019-2026 (2011).
